# Health-related quality of life after epilepsy surgery: A prospective, controlled follow-up on the Iranian population

**DOI:** 10.1038/s41598-019-44442-6

**Published:** 2019-05-27

**Authors:** Mahmoud Lotfinia, Ehsan Nazari Maloumeh, Sina Asaadi, Mahmoud Omidbeigi, Guive Sharifi, Bahador Asadi

**Affiliations:** 1 Doctor of Medicine, Faculty of Medicine, Aja University of Medical Sciences, Tehran, Iran; 2 Skull Base Research Center, Loghman Hakim Hospital, Shahid Beheshti University of Medical Sciences, Tehran, Iran; 3 Department of Neurology, Medical University of Aja, Tehran, Iran

**Keywords:** Epilepsy, Quality of life, Epilepsy

## Abstract

Quality of life is affected by factors such as regional differences in access to treatment choices, and rehabilitation. This study aims to assess the result of epilepsy surgery and its impact on QoL in Iran. The data for 60 patients who underwent epilepsy surgery in Loghman-Hakim hospital between 2003 to 2017 were analyzed prospectively through clinical observation. Clinical variables of interest and the WHOQOL-BREF scale to assess QoL were applied. Scores of operated patients were compared to their preoperative scores as well as epileptic patients controlled with antiepileptic drugs (AEDs) and healthy individuals. The mean age of surgery group patients was 33.78 (34 male; 26 female). Twenty seven patients underwent temporal mesial lobectomy, 20 anterior callosotomy, and 13 neocortical resections. The average QoL score in healthy group was 72.48, in AEDs controls was 56.16, and in operated patients was 65.61. In addition, analysis showed a significant increase in postoperative QoL of the surgical group compared to the AEDs controls. Epilepsy surgery could be the best approach in patients suffering from drug-resistant epilepsy even in developing countries, which can result in seizure relief and a reduction in the frequency of disabling seizures.

## Introduction

Epilepsy is one of the most common diseases of the central nervous system and represents 0.7% of the global burden of disease^1^. According to the World health organization (WHO) reports, about 50 million people worldwide have epilepsy. The frequency of epilepsy in developing countries is much higher^2^; with an approximate prevalence of 15.4 per 1000 compared to 5.8 per 1000 in developed countries^3^. The overall prevalence of epilepsy in Iran is reported to be 5%^4^. Despite the maximum doses of anti-epileptic drugs (AEDs), about one-third of epileptic patients still experience frequent episodes of seizures^5^. The prevalence of refractory epilepsy is about 5 to 8 cases per 1000 persons^6,7^. According to Iranian Epilepsy Association, of the 80000 registered epileptic patients by 2007, 25000 were drug-resistant^8^.

To achieve better control of seizures which are not controlled by AEDs, other therapeutic options such as ketogenic regimens, deep brain stimulation, responsive neurostimulation, and epilepsy surgery were introduced. Nonetheless, about one-third to half of the patients are eligible for epilepsy surgery as the main therapeutic approach for chronic, drug-resistant epilepsy^9–14^. Surgical treatment of refractory epilepsy is a well established therapeutic approach which has been successful in controlling seizure episodes. Clinicians assess the effectiveness of a therapeutic option by how well it has eliminated the patients’ symptoms and complaints, while patients consider how the treatment has affected their daily life. A useful method to assess patients’ satisfaction with treatment is quality of life (QoL), which reflects subjective aspects of the physical, mental, and social life. However one must bear in mind when evaluating a treatment such as epilepsy surgery, that QoL is also affected by factors such as regional variations in access to rehabilitation^15^, treatment choices^16^, comorbid conditions and social supports^17^ worldwide. To our knowledge, there is no previous study about QoL as a tool for evaluating the advantages of epilepsy surgery over other treatment options available in Iran.

The neurosurgery department of Loghman Hakim hospital is one of the most important centers in Iran in which epilepsy surgery is commonly performed. This study aims to assess the results of epilepsy surgery and its impact on health-related quality of life in different aspects such as the mental, physical, living environment and social domains in patients with epilepsy. Since literature lacked a study comparing QoL in patients treated for epilepsy either by surgery or medication in Iran, the present study was designed to perform this QoL comparison between medically controlled patients and healthy controls.

## Results

### Characteristics of the study population

Among the patients undergoing epilepsy surgery in Loghman Hakim hospital between the years of 2003 to 2017, 60 were included in the study. Characteristics of the patients and healthy individuals are summarized in Table 1.

**Table 1:** Preoperative demographic data. ns, non-significant; s, significant.

Variable	Operated patients	Non-operated patients taking AEDs	Healthy group	P-Value
Male N (%)	34 (56.7)	32 (53.3)	30 (50)	
Female N (%)	26 (43.3)	28 (46.7)	30 (50)	
Mean age (year)	33.78 ± 10.98	34.67 ± 9.71	32.03 ± 11.80	0.404 (ns)
Epilepsy duration (year)	11.60 ± 5.96	9.05 ± 5.48	—	0.016 (s)*
Seizure frequency	151.41 ± 131.08	83.73 ± 46.43	—	0.006 (s)*
Seizure type	**N (%)**	**N (%)**		
Partial seizure	28 (46.7)	33 (55)	—	
Complex-partial seizure	10 (16.7)	7 (11.7)		
Generalized seizure	22 (36.7)	20 (33.3)		
Education	**N (%)**	**N (%)**	**N (%)**	
Illiterate	6 (10)	5 (8.3)	3 (5)	0.421 (ns)
Primary education High school diploma	8 (13.3)	13 (21.7)	10 (16.7)	
Bachelor	19 (31.7)	20 (33.3)	20 (33.3)	
Master	12 (20)	13 (21.7)	15 (25)	
PhD	10 (16.7)	13 (21.7)	7 (11.7)	
	5 (8.3)	3 (5)	5 (8.3)	

Based on preoperative assessments, epilepsy surgery was performed in 42 patients (70%) in order to completely eliminate the epileptic seizures (curative surgery) and also in 18 patients (30%) with the purpose of relieving the symptoms as well as enhancing the patients’ QoL (palliative surgery). Twenty seven patients (45%) underwent temporal mesial lobectomy and 20 (33.3%) underwent anterior callosotomy. In 13 patients (21.7%), neocortical resection was performed that 7 out of them had bilateral temporal lesion, 3 had unilateral temporal lesion with extension to extra-temporal regions, 2 had multifocal lesion and 1 patient showed both temporal and extra-temporal lesions (Table 2).

**Table 2:** Surgical outcome based on surgery type.

Engel’s classification	Number of patients undergoing temporal mesial lobectomy (%)	Number of patients undergoing anterior corpus callosotomy	Number of patients undergoing neocortical resection (%)	Total number of patients irrespective of seizure- and surgery type (%)
**Class I**	16 (59.2)	10 (50)	5 (38.4)	31 (51.7)
**A**	8	8	3	18
**B**	3	1	1	3
**C**	5	1	1	9
**D**	0	0	0	1
**Class II**	6 (22.2)	6 (30)	3 (23)	15 (25)
**A**	4	3	2	8
**B**	1	1	1	3
**C**	1	1	0	2
**D**	0	1	0	2
**Class III**	2 (2.7)	3 (15)	4 (30.7)	9 (15)
**A**	2	3	4	9
**Class IV**	3 (11.1)	1 (5)	1 (7.6)	5 (8.3)
**A**	2	1	1	4
**B**	1	0	0	1

### Surgical outcomes

To evaluate surgery outcomes, we used Engel’s classification system as described in Table 3.

**Table 3:** Engel’s classification of epilepsy surgery outcome.

Class I: Free of disabling seizures
A Completely seizure free since surgery
B Nondisabling simple partial seizure only since surgery
C Some disabling seizures after surgery but free of disabling seizures at least for two years
D Generalized convulsions with antiepileptic drug withdrawal only
Class II: Rare disabling seizures
A Initially free of disabling seizures but has rare seizures now
B Rare disabling seizures since surgery
C More than rare disabling seizures after surgery, but rare seizures for at least two years
D Nocturnal seizures only
Class III: Worthwhile improvement
A Worthwhile seizure reduction
B Prolonged seizure-free intervals amounting to greater than half the follow-up period, but not
less than two years
Class IV: No worthwhile improvement
A Significant seizure reduction
B No appreciable change
C Seizures worse

#### Surgical outcomes with regard to patients’ gender

To assess the effect of patients’ gender on the surgery outcomes, Fisher exact test was employed which showed no significant increase or decrease in surgical outcome between the males and females of the study population (P = 0.446).

#### Surgical outcomes in patients with partial seizure

Among 28 patients with partial seizure, 14 (50%) were eligible for class I of Engel’s classification (Table 3), 9 (32.1%) for class II, 3 (10.7%) for class III and 2 (7.1%) for class IV, when assessing surgery outcomes. Among the 14 patients of class I, 7 were well qualified for subclass IA, 2 for subclass IB and 5 for subclass IC. Among those of class II, 6 were highly qualified for subclass IIA, 1 for subclass IIB, 1 for subclass IIC and 1 for subclass IID. All the patients of Class III were eligible for subclass IIIA and among those of class IV, 1 was qualified for subclass IVA and the other one for subclass IVB (Table 4).

**Table 4:** Surgical outcome based on seizure type.

Engel’s classification	Number of patients with partial seizure (%)	Number of patients with complex-partial seizure (%)	Number of patients with generalized seizure (%)	Total number of patients irrespective of seizure- and surgery type (%)
**Class I**	14 (50)	7 (70)	10 (45.4)	31 (51.7)
**A**	7	5	6	18
**B**	2	1	0	3
**C**	5	1	3	9
**D**	0	0	1	1
**Class II**	9 (32.1)	1 (10)	5 (22.7)	15 (25)
**A**	6	0	2	8
**B**	1	1	1	3
**C**	1	0	1	2
**D**	1	0	1	2
**Class III**	3 (10.7)	1 (10)	5 (22.7)	9 (15)
**A**	3	1	5	9
**Class IV**	2 (7.1)	1 (10)	2 (9)	5 (8.3)
**A**	1	1	2	4
**B**	1	0	0	1

#### Surgical outcomes in patients with complex-partial seizure (CPS)

Among 10 patients with CPS, 7 (70%) were classified as class I according to Engel’s classification (Tables 3), 1 (10%) as class II, 1 (10%) as class III and 1 (10%) as class IV. Among those of class I, 5 were categorized as subclass IA, 1 as subclass IB and 1 as subclass IC. All patients of classes 2, 3 and 4 were eligible for subclasses IIB, IIIA and IVA, respectively (Table 4).

#### Surgical outcomes in patients with generalized seizure (GS)

Among 22 patients suffering from GS, 10 (45.4%) were qualified for class I of Engel’s classification (Tables 3), 5 (22.7%) patients were eligible for class II, 5 (22.7%) for class III and 2 (9%) for class IV. Among the patients of class I, 6 were classified as subclass IA, 3 as subclass IC and 1 as subclass ID. Among those of class II, 2 were categorized as IIA, 1 as IIB, 1 as IIC and 1 as IID. All patients of classes 3 and 4 were qualified for subclasses IIIA and IVA, respectively. The surgery outcomes based on seizure type are summarized in Table 4.

Fisher exact test was employed between all three groups to clarify whether the type of seizure could be a prognostic factor for surgical outcome (Table 5).

**Table 5:** Analysis of seizure outcome comparing class I and classes II–IV.

Variable	Class I	Classes II–IV	P Value
Number of patients	31	29	
Gender, M/F*	16/15	18/11	0.446 (ns)
Age A/C	27/4	27/2	0.672 (ns)
SeT, PS/CPS	14/7	14/3	0.460 (ns)
SeT, PS/GS	14/10	14/12	0.783 (ns)
SeT, CPS/GS	7/10	3/12	0.265 (ns)
SuT AC/MTL	10/16	10/11	0.566 (ns)
Sut AC/NLR	10/5	10/8	0.722 (ns)
SuT MTL/NLR	16/5	11/8	0.314 (ns)

Class I = seizure free; Class II = rare seizure; Class III = meaningful seizure improvement; Class IV = no seizure improvement; SeT, seizure type; A, adult; C, child; PS, partial seizure; CPS, complex partial seizure; GS, generalized seizure; SuT, surgery type; AC, anterior callosotomy; MTL, mesial temporal lobectomy; NLR, neocortical lesion resection, ns, non-significan * Gender: M = male/F = female.

### Surgical outcomes with regard to the type of surgery

Among 20 patients undergoing anterior callosotomy, 10 (50%) were eligible for class I of Engel’s classification system, 6 (30%) for class II, 3 (15%) for class III and 1 (5%) for class IV. Among 27 patients undergoing temporal mesial lobectomy, 16 (59.2%) were well qualified for class I of Engel’s classification system, 6 (22.2%) for class II, 2 (7.4%) for class III and 3 (11.1%) for class IV. Among 13 patients who underwent neocortical resection, 5 (38.4%) were classified as class I of Engel’s classification, 3 (23%) as class II, 4 (30.7%) as class III and 1 (7.6%) as class IV (Table 2).

Fisher exact test was used between all three groups to clarify the probable effect of surgery type on the surgical outcomes (Table 5). The surgery outcomes based on surgery type are summarized in Table 2.

### Surgical outcomes in all operated patients irrespective of the type of seizure and type of surgery

Irrespective of the seizure and surgery type, 31 patients (51.7%) were classified as class I based on Engel’s classification (Table 3), 15 (25%) as class II, 9 (15%) as class III and 5 (8.3%) as class IV (Table 4). Thus, the majority of patients were qualified for class I of Engel’s classification, while a minority of them was eligible for class IV. As regards subclasses, 18 patients (30%) were classified as subclass IA, 9 (15%) as IC, 9 (15%) as IIIA, 8 (13.3%) as IIA, 4 as IVA, 3 (5%) as IB, 3 (5%) as IIB, 2 (3.3%) as IIC, 2 (3.3%) as IID, 1 (1.7%) as IVB and 1 (1.7%) as ID.

### Surgical complications

The complications caused by epilepsy surgery could be categorized into two groups including neurological and non-neurological complications. The most common non-neurological complications, which were all transient, included infection of the incision site (5%), surgery site hematoma (5%) and brain edema necessitating re-craniotomy (3.3%). Neurological complications were classified as two minor and major groups. The rate of minor complications was 15% including transient speech impairment (5%), transient paresis of cranial nerves (3.3%), transient memory deficit (3.3%), mutism (1.7%) and transient disconnection syndrome (1.7%). The rate of major complication which was also 1.7% included permanent hemiparesis (Table 6).

**Table 6:** Surgical complications.

Type of complication	Frequency
	(N)
**Non-neurological**	
Incision site infection	3
Surgery site hematoma	3
Brain edema necessating re-craniotomy	2
**Neurological**	
Major	1
Permanent Hemiparesis	1
Minor	9
Transient speech impairment	3
Transient cranial nerve paresis	2
Transient memory deficit	2
Mutism	1
Transient disconnection syndrome	1

### Quality of life

#### Results of QoL assessment

**Figure 1:**
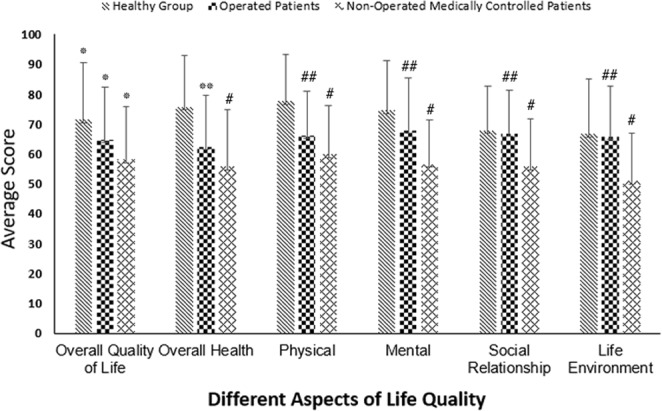
Assessment and comparison of life quality in all three included groups. ^*^P < 0.001 compare to other two groups. ^**^P < 0.05 compare to other two groups. ^#^P < 0.001 compare to healthy group. ^##^P < 0.001 compare to medically controlled patients.

There was no significant difference in the first and second time evaluation of QoL scores between the healthy and medically controlled group (P > 0.05). We decided to report only the result of the second time for statistical analysis of the study. The average QoL score in the healthy group was 72.48 ± 8.12, in patients with epilepsy receiving AEDs was 56.16 ± 6.38 and in operated patients was 65.61 ± 7.36. The mean score was obtained from questionnaire completed by all the study population indicating that epilepsy surgery could enhance the QoL of patients, as the operated patients had a much better QoL than those with epilepsy receiving AEDs only. To determine if the difference in the mean QoL score of all three groups was statistically significant, with assumptions of equal variances based on the result of Levene’s test, One-way ANOVA test was used which showed a significant difference in QoL of the groups (P = 0.000, F (2, 177) = 75.06). LSD post hoc test was also employed, demonstrating that each group showed significant difference in comparison with other groups in the level of QoL (P = 0.000) (Fig. 1).

#### Results of the QoL assessment with regard to five domains included in the questionnaire

As described previously, in world health organization (WHO) quality of life questionnaire, the different domains of QoL are separately evaluated in addition to general assessment of the level of overall QoL. The result of each domain assessment is separately presented in Fig. 1.

#### Satisfaction with life and the own health

In questions 1 and 2, all three groups were asked about their satisfaction with life and their own health condition. In the healthy group, the mean score of the first question was 71.5 ± 19.07, in patients with epilepsy taking AEDs was 58.1 ± 17.7 and in operated patients was 64.75 ± 17.5. The operated patients showed a statistically significant difference compared to the other two groups (P = 0.043 and P = 0.046, respectively). There was also a significant difference between the patients taking AEDs and the healthy group (P = 0.000). The average score of the second question was 75.66 ± 17.42 in healthy group, 55.75 ± 18.92 in patients taking AEDs and 62.33 ± 17.28 in the operated patients. We found a significant difference between the operated patients and those taking AEDs as well as the healthy group (P = 0.045 and P = 0.000, respectively). The patients receiving AEDs also showed a significant decrease compared to the healthy group (P = 0.000). Thus, it could be concluded that the operated patients would have a higher level of QoL compared to those taking AEDs (Fig. 1).

#### Satisfaction with the physical condition

The average score of satisfaction with the physical condition was 78.66 ± 15.49 in the healthy group, 59.83 ± 16.18 in patients receiving AEDs and 66 ± 14.69 in operated patients. A statistically significant difference was found between the operated patients and the healthy group as well as those taking AEDs (P = 0.000 and P = 0.031, respectively). The patients receiving AEDs also showed a significant decrease compared to the healthy group (P = 0.000). Therefore, it is highly possible that the operated patients have a better QoL related to this domain compared to that of patients receiving AEDs (Fig. 1).

#### Satisfaction with the mental condition

The mean score of satisfaction with the mental condition was 74.5 ± 16.61 in the healthy group, 56.58 ± 14.88 in the patients receiving AEDs and 67.83 ± 17.42 in the operated patients. A statistically significant difference was found between the operated patients and the healthy group as well as those taking AEDs (P = 0.000 and P = 0.027, respectively). The patients receiving AEDs also showed a significant decrease compared to the healthy group (P = 0.000) (Fig. 1).

#### Satisfaction with the social condition

In the healthy group, the mean score of satisfaction with the social condition was 67.83 ± 14.99, in the patients taking AEDs 55.75 ± 15.88 and in operated patients 66.83 ± 14.61. There was no statistically significant difference between the operated patients and the healthy group (P = 0.719); however, we found a significant difference between the operated patients and those receiving AEDs (P = 0.000). The patients taking AEDs also showed a significant decrease compared to the healthy group (P = 0.000) (Fig. 1).

#### Satisfaction with the life environment

The last domain of world health organization (WHO) quality of life questionnaire is the life environment assessment. The average score of this domain of QoL in the healthy group was 66.74 ± 18.22, in patients taking AEDs 51 ± 15.96 and in the operated patients 65.91 ± 16.73. We found no statistically significant difference between the operated patients and the healthy group (P = 0.789); however, there was a significant difference between the operated patients and those receiving AEDs (P = 0.000). The healthy group also showed a marked difference in this domain of QoL compared to the patients receiving AEDs (P = 0.000). Thus, epilepsy surgery could bring the patients great satisfaction with this domain of QoL (Fig. 1).

#### QoL at baseline and follow-up within the operated patients

To compare the QoL scores of the operated patients with their own baseline scores, a paired sample t-test was conducted. There was a significant difference in the average QoL scores between baseline condition (M = 56.97, SD = 10.39) and postoperative stage (M = 65.61, SD = 7.36); t (59) = 5.95, p ≤ 0.05. Different domains of reported QoL are summarized in Table 7.

**Table 7:** Reported baseline and postoperative QoL values. s, significant; QoL, quality of life.

QoL domain	Baseline value Mean ± SD	Postoperative value mean ± SD	Paired differences Mean	P-Value
Overall QoL	55.41 ± 18.45	64.75 ± 17.50	9.33	0.004 (s)
Overall Health	54.25 ± 21.26	62.33 ± 17.28	8.08	0.031 (s)
Physical	59.08 ± 19.07	66.00 ± 14.69	6.91	0.023 (s)
Mental	58.33 ± 20.16	67.83 ± 17.42	9.50	0.007 (s)
Social Relationship	57.25 ± 18.18	66.83 ± 14.61	9.58	0.002 (s)
Life Environment	57.50 ± 20.01	65.91 ± 16.73	8.41	0.020 (s)
Average QoL	56.97 ± 10.39	65.61 ± 7.36	8.63	0.000 (s)

## Discussion

About 40 million people with epilepsy live in developing countries^18^, where the condition remains largely untreated because of limited resources, lack of essential drugs, and psychosocial consequences of stigma about their condition^19^. Based on the literature, it is clear that good outcomes for epilepsy surgery can be achieved in low- and middle-income countries^20^. However, outcomes vary based on epilepsy type, expertise, and equipment employed during surgery. Iran is a middle-income country in Western Asia with over 81 million inhabitants. Sayehmiri *et al*.^4^ estimated the prevalence of epilepsy in Iran to be approximately 5% which is similar to the result of other developing countries. Reports demonstrate that the poor awareness of epilepsy surgery among physicians, the lack of health-care infrastructure^21^ and neurosurgeons^22^ are the main reasons that make epilepsy surgery only a theoretical option in Iran. In this study, we have reported the results of different epilepsy surgery methods and their impact on QoL through prospective, controlled follow-up in Iranian patients with epilepsy. The study included the patients that underwent temporal mesial lobectomy, lesionectomy, or anterior callosotomy in Loghman-Hakim hospital between 2003 and 2017. The postoperative outcome was assessed according to the ILAE classification^23^, and WHOQOL- BREF. Comparison of the efficacy of different therapeutic approaches to each other, particularly to those introduced recently such as neuromodulation and selective ablation using laser-thermal technology is beyond the scope of this study; however, these advanced methods are being recently employed in our department which could be the basis of larger studies in the future.

The results of the study showed that 51.7% of the operated patients suffering from drug-resistant epilepsy were seizure-free following the surgery and 24% of them showed a dramatic reduction in disabling seizures (Table 4). Furthermore, postoperative electroencephalograms (EEGs) indicated improvement in every forty-two patients with therapeutic intention. In some studies, it has been suggested that generalized seizure can be a predictive factor for poor outcome of surgery^24,25^, while some other studies reported that surgical outcome would not be affected by generalized seizures^26–28^. Seizure types and surgical outcome were not related in some other reports^29,30^. The most current surgical procedure in our study was mesial temporal lobectomy performed in 27 patients leading to complete freedom of disabling seizures in the majority of them. In addition, anterior corpus callosotomy was the second common surgical procedure resulting in seizure freedom in half of the patients undergoing this type of surgery. We found that despite the more number of seizure-free patients with mesial temporal lobectomy, the statistical analysis showed no significant difference between various surgical approaches in terms of surgery result (Table 2). This is in agreement with some other studies in which it has been suggested that the surgery site is not associated with the surgical outcome^31–33^.

Mere reduction or control of seizure episodes cannot determine the success of a therapeutic approach for establishing a new treatment program in a middle-income country. What patients and clinicians expect from treatment should also be brought into account when evaluating the success of therapeutic approaches. Therefore, in order to evaluate treatment outcomes, different aspects must be considered comprehensively. QoL is a useful method to assess this. QoL is most likely to improve by seizure reduction, though unexpected effects of surgery may contribute to poor QoL despite the reduction in seizure frequency^34–39^. There are some recent studies on the positive impact of epilepsy surgery on QoL, assessed with various questionnaires^40,41^. In this study; we used a generic QoL instrument in our study because when the study started, it was the only test that was validated in Persian. Previous researches have shown that disease-specific instruments are more sensitive to change than generic instruments. Therefore, we expect that the use of a disease-specific QoL survey will increase detecting change over time among the domains. Employing such evaluating tools can help determine success rates of treatment especially in a developing country. In the present study, the QoL of the healthy group members was better than that of the operated patients whose QoL was however higher compared to that of non-operated patients with epilepsy taking AEDs. The difference in QoL between the operated and non-operated patients could be due to lower seizure frequency in the operated patients as mentioned in another study^42^. Furthermore, reduction in the number and dosage of AEDs taken by the operated patients following surgery and also reducing the constraints causing the patients with epilepsy not to be able to participate in different sports and social activities by performing surgery would be the other causes of a higher QoL in the operated patients. Nevertheless, in some studies, it has been mentioned that the QoL of the patients with epilepsy would be improved only with the complete achievement of seizure freedom^43,44^. The small group of included patients and the short term follow up in these studies necessitate more prospective studies to determine the probable causes.

The difference in QoL between the healthy group and the operated patients was significant in all domains of QoL, except in social and life environment domains, while the non-operated patients had lower scores in all domains of QoL compared to the operated patients. Therefore, it could be concluded that the operated patients would have similar compatibility with the life environment and the same ability as the healthy group to establish personal and social contact following surgery since both groups were the same in social and life environment domains of QoL. Moreover, epilepsy surgery would make patients with drug-resistant epilepsy to have a higher QoL as reported by Shi-Yong Liu *et al*.^45^ Assessing the results of 20 studies in a systematic review study demonstrates the important role of surgery in the improvement of QoL of patients with drug-resistant epilepsy. Most review studies on epilepsy surgery outcomes in developing countries point to its efficiency in these nations. Asadi-Pooya *et al*. assert that selecting the best candidates based on the available resources and technologies make epilepsy surgery feasible and cost-effective especially in developing countries^46^ that Espinosa-Jovel *et al*. have expressed it in their new review about epidemiological profile of epilepsy in developing countries^47^. Another interesting finding of this study, that is the first report in Iran, is the increased QoL of the patients in all domains of life quality assessment after the surgery which was obtained from a comparison of given scores of the patients, pre- and postoperation. This finding strongly suggests that epilepsy surgery, if properly chosen and performed, can leave the patient with significantly more quality of life in Iran as a developing country.

Reviewing the complications, no death has been reported peri-operatively or from complications of surgery. The most common non-neurological complications include infection of the incision site, surgery site hematoma, pneumonia and brain edema necessitating re-craniotomy. Medical complications were reported in 6.6% of patients in previous studies^48^. This difference in the incidence of medical complications can be due to limited access to high-quality postoperative care and high prevalence of postsurgical infection in a developing country^49,50^.

The rate of minor neurological complications following surgery was 15% including transient speech impairment, transient paresis of cranial nerves, transient memory deficit, mutism and transient disconnection syndrome. The rate of major neurological complication was 1% including permanent hemiparesis (Table 6). Sindou *et al*.^51^ described the post-surgery complications of 100 operated patients with mesial temporal lobe epilepsy as follows: 3 patients with hematoma, 3 with meningitis, 2 with transient mild hemiparesis and 2 patients needed to undergo ventriculoperitoneal shunt insertion. No mortality was reported in this study. In another study in Canada^52^, 1905 operated patients were included in which no major surgical complication was reported and the rate of incision site infection and hematoma was reported as 1 and 0.7% as a minor surgical complication, respectively. The rate of neurological complication was 3.3% that only 0.5% of the cases showed major neurological complication. Hemiparesis was the most common one and no mortality was reported. The low rate of mortality in the recent studies was obtained after employing microneurosurgery. According to Hader *et al*.^48^ 2013 systematic review to identify studies examining epilepsy surgery complications, minor neurologic complications occurred in 10.9% of operated patients that were twice as frequent in children, whereas major complications were identified in 4.7% of patients. The majority of minor neurological complications are associated with temporal lobe resection, as the effects tend to resolve over time completely^48^. In our study, the rate of transient postoperative complications was higher than that of the studies mentioned above whereas the permanent complications rate was in line with other studies. This difference in the incidence of transient complications can be due to surgical approach because temporal lobe resection was the most commonly used surgical method in our study.

Besides, low socioeconomic level and difficult access to large cities neurosurgical centers^53^ make patients more likely to experience severe symptoms before the first visit. Excessive attention to traditional therapies and lack of educational books and resources about alternative therapies conduce patients not to accept epilepsy surgery therapy at the first step and not to take into account the postoperative rehabilitation recommendations. Insurance coverage limitation for some diagnostic tests is another reason for this shortcoming^54^. On the contrary, very few surgeons experienced in epilepsy surgery are trained abroad and these operations only take place in major cities. This workload degrades proper attention to postoperative care.

The limitations of this study should be noted. The sample size is small so a type II error may have been committed. A larger group of patients in every type of surgery is required to adequately evaluate the therapeutic effects. Due to the importance of the surgical outcome, a Wada test was required to look at language and memory function on each side of the brain, which was not possible for this study.

The results of our study revealed that the majority of patients with epilepsy in Iran could experience seizure freedom or dramatic reduction in disabling seizures following epilepsy surgery irrespective of the type of surgery and the type of seizure. We found that patients undergoing epilepsy surgery had a much greater QoL compared to non-operated patients taking only AEDs. Moreover, surgical treatment appeared to be associated with a low rate of morbidity and no mortality, indicating the high efficacy of the surgical procedure in the treatment of patients with epilepsy.

## Materials and Methods

### Study population

In this study, we prospectively recorded the data of all patients who underwent epilepsy surgery in Loghman Hakim hospital from 2003 to 2017. Loghman Hakim hospital is a tertiary referral center and one of the most equipped neurosurgical centers in Iran. The patients diagnosed with localized-related refractory epilepsy with the absence of mental retardation (IQ > 70) and the absence of physical illness were eligible for inclusion in the study. Exclusion criteria included previous epilepsy surgery, neurostimulation, syndromic epilepsy, and neurometabolic disorders. Of the 92 drug-resistant cases of epilepsy operated between 2003 and 2017, 60 patients who had met our inclusion criteria (Table 8) were willing to participate in our study. The study design was approved by the ethics committee of Shahid Beheshti University of Medical Sciences. In the current study, drug-resistant epilepsy is defined as: “failure of adequate trials of at least two tolerated, appropriately chosen and used AEDs schedules (whether as monotherapies or in combination) with an accurate dosage (determined with evaluation of serum level of drugs) to achieve sustained seizure freedom” according to International League Against Epilepsy^55^.

**Table 8:** Selection criteria used in this study.

Criteria list	Operated patients	Non-operated patients taking AEDs	Healthy group
Presence of detailed pre-, post- and Intraoperative data	Yes	No	No
Clinically and para-clinically confirmed seizure	Yes	Yes	No
Seizure induced functional impairment	Yes	No	No
Incidence of drug-resistant epilepsy	Yes	No	No
Seizures lasting at least more than two years	Yes	Yes	No
Constant usage of AEDs more than one year	Yes	Yes	No
Intelligent quotient more than 70	Yes	Yes	Yes
Age between 10 to 55 years old	Yes	Yes	Yes
No prior brain surgery	Yes	Yes	Yes
Cooperative individual in data collection process	Yes	Yes	Yes

Of the epileptic patients referring to Loghman- Hakim Hospital Neurology Clinic whose disease was controlled on AEDs therapy, 60 patients who matched operated group for age, gender and education and were willing to participate in the study were chosen as the group of patients controlled on AEDs. In order to obtain a healthy control group, flyers were disseminated throughout the hospital and 60 of the volunteers who matched the previous two groups of our study for age, gender and education were selected. Participants of the healthy control group could not be family members of epileptic patients. Also, neither these participants nor their family members had a chronic disease, since it could negatively affect their QoL.

### Pre-surgical evaluations

Patients were referred for surgery by an experienced team consisting of a neurosurgeon, an epileptologist and an anesthesiologist. After extensive preoperative evaluation, including a detailed history of the patients and physical examination, video-electroencephalography, brain magnetic resonance imaging (MRI) was performed to exclude any secondary causes as well as confirming the diagnosis. Patients were also assessed for intelligence quotient (IQ) and neuropsychiatric conditions. Cases of hippocampal sclerosis, hippocampus atrophy or any other pathological changes on MRI related to epileptogenic foci were considered as the best candidates for epilepsy surgery. In patients that MRI showed none of the pathological changes mentioned above or demonstrated extra-temporal impaired regions, epileptogenic foci were determined by means of EEGs. In some patients, molecular imaging methods were also employed if needed. For example, patients in whom MRI failed to reveal pathological changes, the presence of focal showing regions evidenced by EEG along with focal hypo-metabolic foci on Single-photon emission computed tomography (SPECT) was considered as possible candidates for surgery. Twenty-six patients (43%) also underwent F-18-fluoro-deoxyglucose positron emission tomography (PET) scan. Nine patients (15%) and three patients (5%), respectively underwent Stereoelectroencephalography and implantation of subdural grids as a diagnostic workup, respectively. Depth electrodes were used in two patients (3%).

In this study, epilepsy surgery is categorized into therapeutic or palliative surgery. In specific situations in which a surgical cure is not possible, Palliative surgery was performed to alleviate patients’ symptoms with the main aim of minimizing the frequency and severity of seizures as much as possible.

### Surgery

All epilepsy surgeries were performed in Loghman Hakim hospital affiliated to Shahid Beheshti University of Medical Sciences. For lesions of the inner surface of the temporal lobe, temporal mesial lobectomy was performed including microsurgical removal of hippocampal and amygdala structures. For lesions of the neocortex, complete lesionectomy was performed, if the lesion was located somewhere other than eloquent cortex and its removal caused no severe disability to the patients. In patients with the history of drop attack without any lesion related to the epileptogenic foci, anterior callosotomy was performed. In this surgical procedure, the patient was positioned supine and an S-shaped incision was made on the right coronal suture region. After craniotomy was performed, dura mater was opened in a C-shape and microdissection was done to reach the corpus callosum using a microscope. Lastly, half or anterior two-thirds of the corpus callosum was sectioned.

### Patients’ follow up, assessment of the surgical outcome and patients’ QoL

The operated patients were followed up monthly in Loghman Hakim hospital for a minimum of 6 months, and the maximum follow-up duration in our study was 48 months. All patients were assessed by experienced neurosurgeons to postoperatively assess seizure frequency and neurological complications of the epilepsy surgery. In addition to the comparison of pre- and postoperative seizure frequency, the frequency of seizures was also evaluated based on Engel’s classification (Table 3) to determine slightest changes in seizure frequency. In order to ensure the outcome of the surgery, postoperative EEG was performed 4–6 months after surgery only in patients with therapeutic intention. Moreover, QoL of the patients was also assessed with the Persian version of the World Health Organization’s QoL Instrument-Short Version (WHOQOL-BREF) which is the only QoL questionnaire that had been validated in Persian^56–58^. WHOQOL- BREF is the shorter version of the WHOQOL-100 quality-of-life assessment. Unfortunately, the sensitivity and specificity of WHOQOL-BREF Persian version have not been reported in epileptic patients, but the internal consistency reliability was 0.8406 and the validity was 0.6516 by comparison to WHOQOL-100 in Thai population. The questionnaire is comprised of 26 questions: 24 questions covering four aspects including physical and mental health, social relationships and environment: two additional questions assess general satisfaction with health status and quality of life. The first two questions were related to the personal satisfaction of patients, questions 3 to 9 were related to the physical domain, questions 10 to 15 were related to the mental domain, and questions 16 to 18 and 19 to 26 were also related to social and life environment domains, respectively. The score of each domain ranged from 0 to 100, and the higher scores were associated with a better QoL. Patients were asked to complete this questionnaire one week prior to the surgery as a part of the preoperative evaluation (baseline or preoperative QoL) and once during the 18th to the 24th-month visit. In case of low level of education for reading the questions, physical disabilities, etc., a physician (EN) would assist in filling it. For those patients who could not come in for filling the questionnaire, the questionnaires were exchanged via post.

The operated patients completed the questionnaire, and the results were collected. Sixty patients receiving AEDs and sixty healthy controls were also included in the study to complete the questionnaire twice, once at the same time with the pre-surgical and once about with post-surgical survey of the surgical group to compare the quality of life of all three groups.

### Statistical analysis

In the present study, the sample size was calculated by Chi-square test in NCSS software in order to have 80% power, freedom degree 2.5 and effect size 0.5. The intended sample size was 55 for each group, of which 60 patients were included to increase the accuracy. Data analysis was conducted using SPSS Statistics Version 22. Shapiro-Wilk test was used to assess the normalization of patients’ distribution. Additionally, Fisher exact test was employed to evaluate the effect of gender, age group, type of seizure and type of surgery on the surgical outcome. The QoL scores of all three groups including operated patients, patients with epilepsy controlled with medical treatment and the healthy controls were recorded. Patients in the operated group were assessed for QoL pre- and post-surgically. The QoL scores of all three groups were compared using One-way ANOVA test. Also, LSD post hoc test was employed to demonstrate whether each group showed a significant difference in comparison with other groups. A P-value < 0.05% was considered statistically significant. Comparisons between baseline and follow-up scores were conducted using the paired sample t-test.

### Ethical approval

All procedures performed in studies involving human participants were in accordance with the ethical standards of the institutional and national research committee (ethics committee of Shahid Beheshti University of Medical Sciences) and with the 1964 Helsinki declaration and its later amendments or comparable ethical standards. Informed consent was obtained from all individual participants included in the study.
